# Decoding aptamer-protein binding kinetics for continuous biosensing using single-molecule techniques

**DOI:** 10.1126/sciadv.ads9687

**Published:** 2025-02-14

**Authors:** Mike Filius, Lena Fasching, Raman van Wee, Alina Y. Rwei, Chirlmin Joo

**Affiliations:** ^1^ Department of BioNanoScience, Kavli Institute of Nanoscience, Delft University of Technology, 2629 HZ Delft, Netherlands.; ^2^ Department of Chemical Engineering, Delft University of Technology, 2629 HZ Delft, Netherlands.; ^3^ Department of Physics, Ewha Womans University, Seoul 03760, Republic of Korea.

## Abstract

Continuous biosensing provides real-time information about biochemical processes and holds great potential for health monitoring. Aptamers have emerged as promising alternatives over traditional biorecognition elements. However, the underlying aptamer-target binding interactions are often poorly understood. Here, we present a technique that can decode aptamer-protein binding interactions at the single-molecule level. We demonstrate that our single-molecule assay is able to decode the underlying binding kinetics of aptamers despite their similar binding affinity. Guided by computational simulations and validated with quartz crystal microbalance experiments, we show that the quantitative insights generated by this single-molecule technique enabled the rational understanding of biosensor performance (i.e., the sensitivity and limit of detection). This capability was demonstrated with thrombin as the analyte and the structurally similar aptamers HD1, RE31, and NU172 as the biorecognition elements. This work decodes aptamer-protein interactions with high temporal resolution, paving the way for the rational design of aptamer-based biosensors.

## Teaser

Single-molecule techniques reveal how aptamer-protein binding kinetics affect the sensitivity and limits of continuous biosensors.

## INTRODUCTION

Biosensors have garnered substantial interest in recent decades due to their ability to detect and quantify biomolecules, playing a key role in health monitoring and disease detection (*1*). As biological systems undergo constant changes, real-time and continuous biosensing is required to capture time-dependent biological processes (*1*, *2*). To achieve this, it is vital to develop highly sensitive, selective, and regenerable biorecognition elements (*3*).

Aptamers are short, single-stranded sequences designed to bind a specific target epitope on a target compound, often a protein (*4*). Typically, aptamers are composed of nucleic acids and have a self-complementary region, allowing them to fold into secondary structures. Aside from their usage as a tool in molecular biology, aptamers are also being used in a clinical setting for therapeutic and diagnostic purposes. In biosensing applications, aptamers have emerged as a promising alternative to the gold standard antibodies and enzymes, due to their simple and cost-effective production and modification, high binding affinities, and high selectivity (*2*). In addition, the ability to regenerate and reuse aptamers without substantial loss of performance is a key advantage over traditional biorecognition elements, such as antibodies (*5*). Consequently, this has led to the development of numerous aptamer-based biosensors that are targeting a variety of analytes, ranging from small molecules (*6*) to proteins (*7*) and even entire cells (*8*), demonstrating the high and diverse applicability aptamers in the sensing field.

Despite the large number of aptamers developed, the cases where aptamers are successfully used in sensor systems remain limited (*9*, *10*). Currently, the selection of aptamers for biosensing applications is mostly done based on the affinity value (e.g., dissociation constant, *K*_d_) of aptamer-target interactions that are derived from their selection procedures or post-selection binding studies. The aptamer-target interactions have been investigated with biophysical approaches such as circular dichroism spectroscopy, isothermal calorimetry, nuclear magnetic resonance spectroscopy, and x-ray crystallography (*11*). These methods, however, provide ensemble average measurements (i.e., a signal is generated by a collection of molecules) and suffer from several intrinsic limitations, namely, high sample consumption, restricted sensitivity, and the masking of subpopulation heterogeneity. Surface plasmon resonance can overcome these limitations but is labor intensive and kinetic measurements are distorted by mass transport effects and surface heterogeneity (*12*). Thus, to accurately quantify the aptamer-target interactions, advanced sensing and analysis modalities are needed.

Here, we present a single-molecule fluorescence platform to quantify aptamer-protein interactions with high temporal resolution. In our platform, individual proteins are immobilized on a surface and the binding of fluorescently labeled aptamers is detected in real time through total internal reflection (TIR) microscopy. For our proof-of-concept experiments, we selected the thrombin-aptamer pair due to the availability of multiple aptamers, providing a stringent test for our platform (*13*, *14*). We challenged our single-molecule fluorescence resonance energy transfer (smFRET) assay to characterize three structurally similar aptamers, HD1, RE31, and NU172, that bind to the same domain (exosite I) on the thrombin target protein (*15*, *16*). We demonstrate that our platform can robustly quantify the association (*k*_on_) and dissociation rate (*k*_off_) as well as the dissociation constant (*K*_d_). We obtained similar dissociation constants for aptamers HD1, NU172, and RE31 but vastly different association and dissociation rate constants for these aptamers, emphasizing that the underlying binding mechanism is different for each aptamer.

To demonstrate the ability for the rational design of an aptamer-based biosensor, we used our single-molecule kinetic information for the development of a mass-based thrombin biosensor using quartz crystal microbalance (QCM). Guided by computational simulations and validated QCM experiments, we show that the sensor performance (i.e., the sensitivity and limit of detection) is not only dependent on the aptamer dissociation constant (*K*_d_), but more importantly on the association (*k*_on_) and dissociation rates (*k*_off_). This study features a single-molecule technique with high temporal resolution to decode the aptamer-target binding kinetics, paving the way for the rational design of aptamer-based biosensors.

## RESULTS

**Fig. 1.**
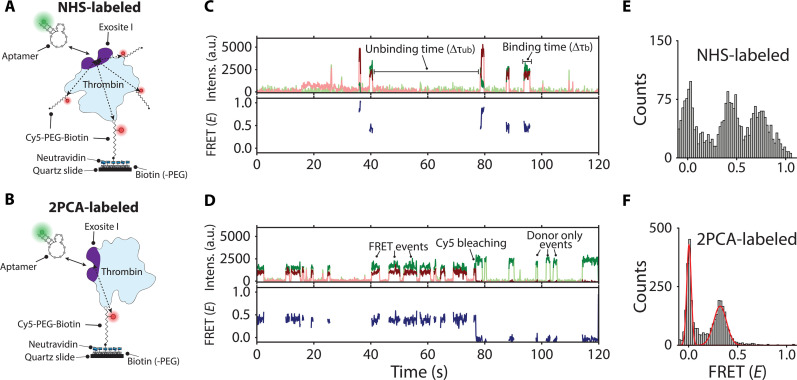
Single-molecule aptamer characterization assay. (**A** and **B**) Schematic representation of the single-molecule FRET assay. The NHS (A) or 2PCA (B) acceptor (Cy5)–labeled thrombin proteins are immobilized on a PEGylated quartz slide through biotin-neutravidin conjugation. Binding of the donor (Cy3)–labeled aptamer to the protein target yields a FRET signal. (**C** and **D**) Representative time traces for NHS-labeled thrombin (C) or 2PCA-labeled thrombin (D). Bleaching of the acceptor (Cy5) fluorophore yields aptamer binding events with donor (Cy3) fluorescent signal only. This yields a “donor only” peak at FRET (*E*) of 0.0 in (D) and (F). (**E** and **F**) Ensemble FRET histograms for all molecules in a single field of view. The FRET distribution is built from the mean FRET efficiency of each binding event. (E) NHS-labeled thrombin yields a heterogeneous FRET histogram. (F) 2PCA-labeled thrombin yields a FRET histogram with a single peak, reporting on the location of the donor-labeled aptamer binding site relative to the N terminus. The FRET histogram is fitted with a Gaussian function, the mean FRET efficiency is 0.38 for aptamer HD1.

Single-molecule fluorescence has been used to quantify the conformational dynamics of aptamers upon ligand biding (*17*, *18*). As these approaches primarily focus on intramolecular conformational changes within the aptamer and do not capture the real-time kinetics of aptamer-ligand interactions, we designed a single-molecule assay where acceptor (Cy5)–labeled thrombin proteins were immobilized on a polymer-coated quartz slide through biotin-neutravidin conjugation. Next, donor (Cy3)–labeled aptamers were injected, and the binding of aptamers to thrombin was monitored using TIR fluorescence microscopy (Fig. 1).

For the development of a robust aptamer-protein characterization assay, we reasoned that fluorescently tagging and immobilization of the target protein should be done at a defined site on the protein (e.g., the N terminus of a protein). This would reduce artifacts arising from immobilization orientation bias or interference from a large number of fluorophores attached near the binding site. For this, we used our recently developed bifunctional linker (*19*) that combines 2-pyridinecarboxaldehyde (2PCA) chemistry (*20*) and copper-free click chemistry to label the N terminus of protein targets. To demonstrate the site-selective fluorescence labeling and protein immobilization, we compared the smFRET data of thrombin labeled with *N*-hydroxysuccinimide sodium salt (NHS)–ester that targets all amines (Fig. 1A) with N-terminal 2PCA-labeled thrombin (Fig. 1B). The assay was designed to give a FRET signal upon specific binding of the donor (Cy3)–labeled aptamer to the immobilized protein. We observed short-lived, repetitive binding events of the aptamer to the target protein, for both NHS-labeled and 2PCA-labeled thrombin (Fig. 1, C and D). These single-molecule time traces represent the direct visualization of transient binding of the HD1 aptamer, explicitly validating previous inference drawn from ensemble data (*21*, *22*). The absence or bleaching of the acceptor (Cy5) fluorophore yields aptamer binding events with donor (Cy3) fluorescent signal only, yielding a “donor only” peak at a FRET (*E*) of 0.0 (Fig. 1, E and F). We obtained a heterogeneous FRET histogram for NHS-labeled thrombin (Fig. 1E) and a defined FRET histogram for 2PCA-labeled thrombin (Fig. 1F). The FRET heterogeneity [FRET (*E*) ~ 0.2 to 0.8] for NHS-labeled thrombin suggests variability in the location of acceptor fluorophores across different protein molecules, likely due to the labeling of different lysine residues in each molecule when the NHS-ester conjugation strategy is used. In contrast, the sharply defined FRET efficiency peak [FRET (*E*) = 0.38 ± 0.14, mean ± SD] for 2PCA-labeled thrombin corresponds to aptamer binding events at a fixed distance from a single reference point—the N terminus. Differences between NHS-labeled and 2PCA-labeled thrombin are further highlighted by the dwell-time analysis of the aptamer bound state (or binding time) and the binding frequency (or unbound time) (fig. S1). We speculate that the NHS-labeling approach has resulted in acceptor fluorophores being conjugated to a lysine in the aptamer/protein binding site, hampering the aptamer interaction with the target protein.

Next, we sought to distinguish the binding kinetics of different thrombin aptamers using our smFRET assay. For this, we selected three aptamers (HD1, RE31, and NU172) that all bind exosite I on thrombin (fig. S2A). Each aptamer has a distinct structure (fig. S2B) and yields different FRET efficiencies reporting on the structural differences of the aptamers relative to the N terminus of thrombin (fig. S2, C and D). The aptamer RE31, analogous to HD1, is stabilized by the addition of duplex domains in the 5′- and 3′-ends of HD1 (*23*). Another similar aptamer, NU172, is stabilized by altering the loops within G-quadruplex in combination with addition of a duplex domain of HD1 (*14*, *24*). We found that *K*_d_ values are lower for RE31 (2.9 ± 0.1 nM) and NU172 (3.3 ± 0.7 nM) compared to that for HD1 (9.2 ± 0.8 nM), agreeing with the literature (table S1).

**Fig. 2.**
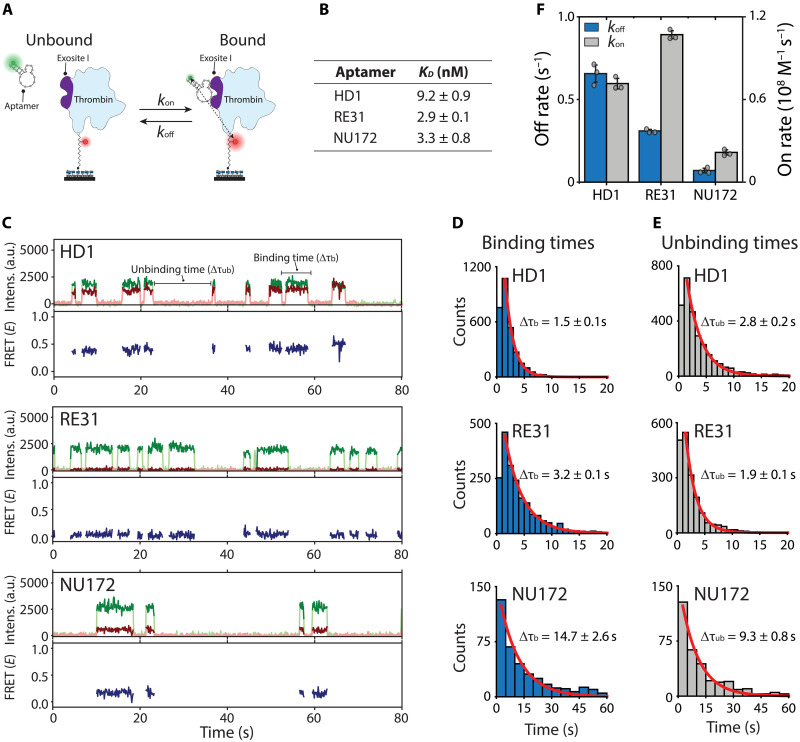
Single-molecule kinetic characterization of the aptamer-protein interactions. (**A**) The association (*k*_on_) and dissociation (*k*_off_) rate can be determined from the dwell times of the aptamer going from the unbound to the bound state. (**B**) The dissociation constant determined as *K*_d_ = *k*_off_/*k*_on_ for each of the aptamers with thrombin. The *K*_d_ is reported as the mean ± SD of at least three independent measurements. (**C**) Representative time traces for HD1 (top), RE31 (middle), and NU172 (bottom). Transient and repetitive binding is observed for each of the aptamers. (**D** and **E**) The binding (τ_b_, D) and unbinding (τ_ub_, E) dwell time distributions are plotted for each of the aptamers and fitted with a single exponential fit. The dwell times are reported as the mean ± SD of three independent experiments. (**F**) Overview of the obtained dissociation-(*k*_off_ = 1/τ_b_, blue bars) and association (1/τ_ub_ = *k*_on_*c*, gray bars) rates for each of the aptamers. The rates are reported as the mean ± SD of three independent experiments.

Our smFRET assay allows us to further decode the underlying binding interactions of the three aptamers by determining the on- and off-rates with thrombin (Fig. 2A). We measured the mean binding time from individual binding events for each of the aptamers (Fig. 2C), from which we could determine the dissociation rate (see the definition of the kinetic rates in Materials and Methods). We observed that the *k*_off_ is at least twice as high for HD1 (*k*_off_ = 0.7 ± 0.1 s^−1^) in comparison to aptamers with a stabilized structure as for RE31 (*k*_off_ = 0.3 ± 0.1 s^−1^) and NU172 (*k*_off_ = 0.1 ± 0.1 s^−1^) (Fig. 2D, blue bars).

The association rate can be calculated from the time between binding events (or unbinding time, τ_ub_, Fig. 2E) (see the definition of the kinetic rates in Materials and Methods). We found the expected faster association rate for RE31 (1.1 ± 0.1 × 10^8^ M^−1^ s^−1^) in comparison to HD1 (0.7 ± 0.1 × 10^8^ M^−1^ s^−1^). However, this was not the case for NU172 (0.2 ± 0.1 × 10^8^ M^−1^ s^−1^) (Fig. 2D, gray bars). This suggests that not only the length of the duplex, but also subtle differences in sequence between RE31 and NU172 (fig. S2B) result in different *k*_off_ and *k*_on_ values of the aptamer-protein interaction. Altogether, the kinetic analysis using our smFRET assay directly observes the transient nature of the interaction and reveals that the underlying binding interaction is different for each of the aptamers.

**Fig. 3.**
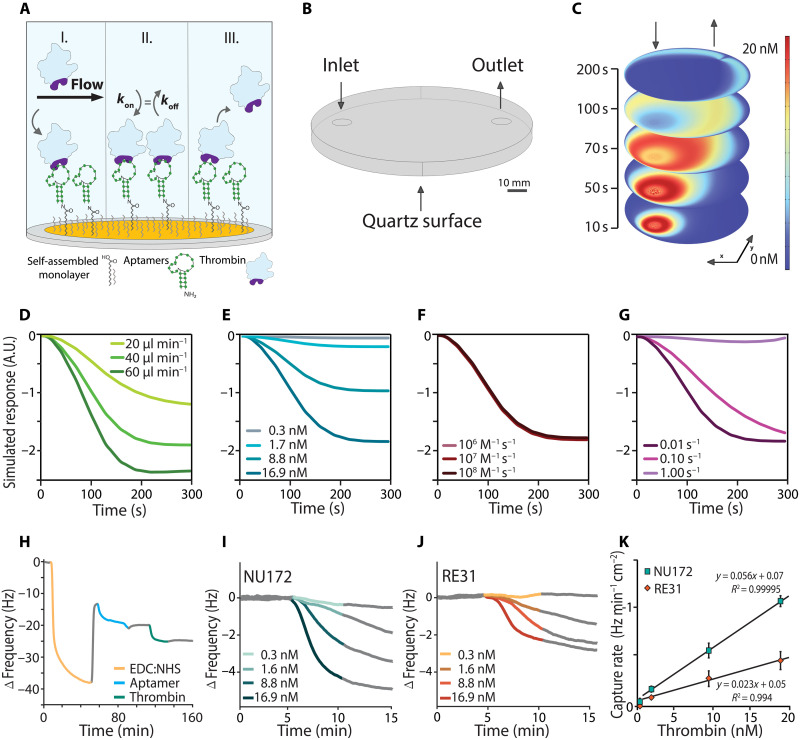
Aptamer-protein interactions in a biosensing application. (**A**) Schematic of a QCM experimental setup and aptamer thrombin capture in different stages of an experiment: (I) association phase, (II) equilibrium state, and (III) dissociation phase. (**B**) Geometry of a QCM measurement chamber used for COMSOL simulations. (**C**) Thrombin concentration distribution at the mid-height of the QCM chamber at different time points during the simulation, considering the effects of laminar flow, diffusion, and convection. (**D** to **G**) Simulation results for various parameters: (D) flow rate, (E) concentration, (F) *k*_on_ rate, and (G) *k*_off_ rate. The plotted curve represents the inverse of the concentration of thrombin-aptamer complexes formed at the bottom boundary of the surface. (**H**) Example plot of a complete QCM measurement including PBS equilibration time and rinsing steps (gray), EDC:NHS activation (yellow), aptamer immobilization (blue), and thrombin capture (turquoise) at the quartz gold surface. (**I** and **J**) QCM target capture results for aptamers NU172 and RE31 at different thrombin concentrations. Frequency reduction within the first 5 min is highlighted, and baseline correction and a moving average noise filter were applied to improve the visibility of the frequency change. (**K**) Sensitivity of the aptamer-based biosensors. The figure shows the sensor’s capture rate with respect to thrombin concentration, the slope of which is the sensor’s sensitivity toward thrombin. The aptamers used were NU172 and RE31 (error bar = SD, *n* = 3).

We sought to use our single-molecule data for the rational selection of an aptamer for a real-time, continuous sensing platform for thrombin. To this end, we used the mass-sensitive QCM as a representative setup for continuous monitoring biosensing platforms (Fig. 3, A and B). The QCM, operated under flow conditions, enables real-time tracking of target capture and dissociation by measuring changes in the quartz’s resonance frequency. These frequency changes are directly related to the added mass on the quartz surface, as described by the Sauerbrey equation (*5*). To investigate the effects of mass transfer on target capture and its subsequent impact on capture kinetics in such a biosensing context, we conducted a COMSOL simulation including fluid flow, diffusion-convection, and the interactions between immobilized aptamers and thrombin at the bottom boundary (Fig. 3, B to G). We simulated the sensor response for 300 s, with the first 100 s allowing thrombin flow to initiate the association phase (phase I, Fig. 3A) and the subsequent water flow initiating the dissociation phase (phase III, Fig. 3A), rather than simulating the signal at equilibrium conditions (phase II, Fig. 3A). This approach, resembling a continuous monitoring platform with rapid sampling times, ensures the capture of dynamic binding and unbinding events in QCM experiments.

We systematically altered the simulation parameters: thrombin concentration, flow rate, *k*_on_, and *k*_off_ rate to assess their effect on the sensor response. Varying the flow rate, we observed that an increased flow rate results in a faster onset of the sensor response (Fig. 3D), showing that the sensor’s response under flow conditions is constrained by mass transport of thrombin to the surface. The simulated sensor response curves show a proportional relationship between thrombin concentration and the rate of aptamer-thrombin complex formation (Fig. 3E).

Variations in the association rate constants (*k*_on_) of aptamer binding did not affect the sensor’s response within the simulation’s time frame (Fig. 3F). Conversely, alterations in the dissociation rate constants (*k*_off_) significantly influenced both the capture rate and dissociation behavior (Fig. 3G). A high *k*_off_ value resulted in drastically reduced sensor response and nearly complete target release within the simulated 300 s, whereas the other tested two lower *k*_off_ values showed no thrombin release, likely due to surface rebinding given the unsaturated surface. To identify the aptamer with the highest sensitivity in such a sensor setup, we ran a COMSOL simulation using the *k*_on_ and *k*_off_ values obtained from smFRET measurements (fig. S3), indicating that NU172 would show higher sensitivity in comparison to RE31 and HD1. While the model incorporates several idealized assumptions about flow and diffusion behavior (see note S1), it effectively indicates the mass transport limitations inherent in such a sensor setup and their impact on the sensor response. Thus, we hypothesized that NU172, the aptamer with the slowest measured *k*_off_, would perform best (i.e., highest sensitivity and lowest limit of detection) in an aptamer-based sensor. To test the validity of the computed performance of the aptamers, we conducted QCM experiments with the respective aptamers under flow conditions (Fig. 3A). We immobilized the amino-modified aptamers on the gold electrodes of the quartz crystal using a self-assembled monolayer with carboxyl groups and *N*-(3-dimethylaminopropyl)-*N*′-ethylcarbodiimide (EDC) activation (fig. S4), along with hydroxy groups for passivation (fig. S9).

We started with quantifying the immobilization efficiency of the different aptamers in our QCM experiments and observed sufficient immobilized aptamers for NU172 and RE31, but not for HD1, making this analysis only possible for NU172 and RE31 (fig. S5). We evaluated the frequency reduction within the first 5 min (i.e., the linear decrease during the association phase, Fig. 3A, I) after introduction of different thrombin concentrations on the immobilized aptamers NU172 and RE31 in our QCM experiments (Fig. 3, I and J). The signal response, i.e., the slope between frequency and time at the linear range upon introduction of thrombin, is proportional to the thrombin concentration in the range of 1.65 to 16.5 nM (Fig. 3, I and J). NU172 exhibits approximately twice the sensitivity of RE31, as indicated by the slope of the linear correlation between rate of frequency decrease and concentration (Fig. 3K). The higher sensitivity of NU172 further validates our COMSOL simulation results. Below this concentration range, RE31 did not show a significant frequency change, while NU172 responded at 300 pM, but the response did not follow a linear correlation with concentration, indicating that it was below the limit of quantification. When we flushed the QCM chamber with phosphate-buffered saline (PBS), we observed thrombin dissociation from the aptamers, which is indicated by a frequency increase in the QCM results (Fig. 3H, gray trace after target capture). The observed dissociation rate from QCM measurements generally follows the expected trend, with RE31 showing higher release rates than NU172 at a thrombin concentration of 16.5 nM (fig. S6). Ensemble-based QCM sensing suggested that RE31 and NU172 demonstrated similar regeneration capabilities when rinsed with water alone, achieving a recovery of 79 ± 2% and 76 ± 8% of the initial signal, respectively. A further optimization in regeneration using the NU172 aptamer improved the recovery efficiency to 89 ± 3% with an 8 M urea buffer and reached 91 ± 7% recovery after using a NaOH regeneration buffer (fig. S6).

Altogether, the QCM experiments agree with the COMSOL simulation results, confirming that under flow conditions and mass-transport limitations, a prolonged lifetime of the aptamer-protein complex leads to more stable sensor responses and a higher sensor performance in analyte sensitivity and limit of detection.

## DISCUSSION

Here, we developed a single-molecule assay to investigate protein-aptamer interactions with high temporal resolution. We demonstrated that our single-molecule assay can differentiate the underlying binding kinetics of aptamers with similar binding affinity, which is crucial for the rational selection of aptamers in biosensor applications.

We exploit the single-molecule sensitivity of our assay to directly visualize the transient nature of the interaction between thrombin and its aptamers HD1, NU172, and RE31, providing quantitative insights into their binding kinetics. Although thrombin’s interaction with aptamers was described previously, prior methods primarily relied on ensemble data, which are well suited for thermodynamic characterization but fail to capture the real-time kinetics of individual binding events. Our single-molecule assay uncovers the full distributions of association and dissociation rates, revealing subtle differences in the binding mechanisms of aptamers with similar affinities (*21*, *22*). As such, these advances make our approach a powerful tool for the rational selection of aptamers with tailored kinetic profiles for biosensor applications, complementing previous single-molecule fluorescence assay where the conformational dynamics of the aptamer itself were investigated (*17*, *18*, *25*).

Our findings revealed that an aptamer with a lower *k*_off_, such as NU172, enhances sensor sensitivity in real-time continuous monitoring platforms, as demonstrated by our COMSOL simulations and confirmed through QCM experiments. This suggests that while affinity (*K*_d_) is a commonly used metric for aptamer selection, the kinetic parameters, particularly *k*_off_, play a more critical role in determining biosensor efficiency, especially under flow conditions. The lower dissociation rates lead to more stable aptamer-protein complexes, which are essential for improving the limit of detection and overall sensitivity of the sensor.

We demonstrated that COMSOL simulations, informed by real-time association and dissociation rates obtained from smFRET experiments, aid in predicting aptamer performance in sensing applications, such as the mass-sensitive QCM experiments under flow conditions. These simulations can provide valuable insights into the kinetic profiles required for various sensing scenarios and serve as a crucial step in optimizing key measurement conditions, such as flow rate, to enhance sensor performance. Despite relying on idealized assumptions and omitting factors like viscosity effects and environmental variability, their integration with careful experimental validation effectively guides the identification of critical parameters for specific sensing applications.

Aptasensor regeneration in QCM experiments are limited by QCM’s ensemble sensing nature, which hinders effective and rapid regeneration due to additional factors such as the rebinding or retention of target molecules on unsaturated regions of the sensor surface. Further improvements in sensor surface assembly to minimize analyte rebinding and retention could significantly enhance regeneration capabilities. Each sensor’s optimal kinetic profile varies depending on the target and specific measurement conditions: A higher *k*_off_ value is beneficial for regeneration in high–target concentration scenarios, while low–target concentration scenarios require higher sensitivity and a very low *k*_off_, necessitating alternative regeneration methods. Alternative regeneration protocols using DNA-denaturing buffers have been shown to improve the reusability of the QCM sensor. These findings suggest that optimization of regeneration methods in ensemble-mode sensors, such as fine-tuning the regeneration time (*26*) and buffer composition (*27*), is required to enhance the reusability of ensemble-based biosensors by facilitating target release and preventing persistent interactions on the sensor surface across multiple cycles.

The site-specific labeling of a protein via its N terminus using the bifunctional 2PCA-DBCO linker was instrumental in achieving precise measurements of binding kinetics that underlie the conclusions of this study. This labeling strategy ensures consistent fluorophore positioning, reducing variability and artifacts in the observed binding kinetics. The dual labeling of both the aptamer and the ligand is beneficial to reduce false positives and for the quantification of aptamer-ligand interactions with high temporal resolution. The fluorescence labeling of biomolecules (e.g., nucleic acids, proteins, or small molecules) has been successfully implemented in a broad range of single-molecule fluorescence experiments (*28*–*30*), and thus, we speculate that our single-molecule assay can be used to quantify binding kinetics for aptamers and other probes that target a variety of biomolecules.

Our bifunctional linker first conjugates DBCO click functionality to the N terminus of proteins using the 2PCA chemistry (*20*), which can then be used to label proteins at a specific site with a broad range of azide-functionalized cargo (e.g., short DNA linkers, polyethylene glycol (PEG) linker, or fluorophores). This approach removes the need for genetic or synthetic tags, thereby simplifying the experimental procedure and opening up opportunities for analysis of proteins from natural sources.

In conclusion, this study features a single-molecule technique with high temporal resolution to decode the aptamer-target binding kinetics. By gaining in-depth knowledge of aptamer binding kinetics through smFRET, this approach facilitates the rational selection of aptamers with tailored binding characteristics. These insights address key challenges that hinder the widespread clinical use of aptamer-based sensors, including their reliability under variable environmental conditions (*31*)—a limitation often linked to the incomplete understanding of their binding mechanisms (*32*). Combining the kinetics insights of smFRET with the computational simulation of sensor setups and the rigorous validation of key assumptions offers valuable guidance for the development of more sensitive and efficient biosensors, with the potential to advance both fundamental research and clinical diagnostics.

## MATERIALS AND METHODS

### N-terminal modification

The 2PCA-DBCO bifunctional linker was synthesized as described before (*19*). In brief, we incubated 100 mM of a 2PCA intermediate [6-(piperazin-1-ylmethyl)-2-pyridinecarboxaldehyde HCl salt; Sigma-Aldrich: 808571] with twofold excess sulfo-NHS-DBCO (Sigma-Aldrich: 762040) and threefold excess of triethylamine in dimethyl sulfoxide (DMSO) for 24 hours at room temperature, while shaking. Next, we quenched the NHS by adding 10-fold excess dimethylamine and incubated for 4 hours at room temperature. The reaction mixture was dried using speed vac and dissolved in DMSO to a concentration of 100 mM 2PCA-DBCO.

The alpha-thrombin protein (Prolytix, HCT-0020) was dissolved in 1× PBS at a concentration of 10 μM. To this solution, we added 400-fold excess of the 2PCA-DBCO linker and incubated for 24 hours at 37°C while shaking. The next day, free 2PCA-DBCO was removed with Zeba Spin desalting columns with a 7-kDa molecular weight cutoff (Thermo Fisher Scientific: 89882) according to the manufacturer’s manual. Next, the protein was labeled with twofold excess Azide-Cy5-biotin (Click Chemistry Tools; CCT1232) and incubated overnight at room temperature (23 ± 1°C). The next day, the samples were purified from excess Cy5-biotin-azide using Zeba Spin desalting columns in 1× PBS.

### NHS labeling of proteins

For NHS labeling, 5 μM thrombin was labeled with 140-fold excess NHS-DBCO (10-fold excess per lysine, Sigma-Aldrich: 762040) in 1× PBS and incubated overnight at room temperature (23 ± 1°C). The next day, the samples were purified from excess NHS-DBCO using Zeba Spin desalting columns in 1× PBS. Next, the DBCO-modified thrombin protein was labeled with 14-fold excess (2-fold excess per lysine) azide-Cy5-biotin and incubated overnight at room temperature (23 ± 1°C). The next day, the samples were purified from excess Cy5-biotin-azide using Zeba Spin desalting columns in 1× PBS.

### Single-molecule setup

All experiments were performed on a custom-built microscope setup. An inverted microscope (IX73, Olympus) with prism-based TIR was used. In combination with a 532-nm diode-pumped solid-state laser (Compass 215M/50 mW, Coherent), a 60× water immersion objective (UPLSAPO60XW, Olympus) was used for the collection of photons from the Cy3 and Cy5 dyes on the surface, after which a 532-nm long-pass filter (LDP01-532RU-25, Semrock) blocks the excitation light. A dichroic mirror (635 dcxr, Chroma) separates the fluorescence signal, which is then projected onto an electron-multiplying (EM)-charge-coupled device (CCD) camera (iXon Ultra, DU-897U-CS0-# BV, Andor Technology). Our pixel size is 107 nm by 107 nm and the complete field of view is 512 pixels × 256 pixels (54.8 μm by 27.4 μm) and contains ±500 molecules. A series of EM-CCD images was recorded using Andor Solis software (v4.32, Andor).

### Single-molecule data acquisition

Single-molecule flow cells were prepared as previously described (*33*, *34*). After assembly of a microfluidic chamber, the slides were incubated with 20 μl of neutravidin (0.1 mg/ml; Thermo Fisher Scientific: 31000) for 2 min. Excess neutravidin was removed with 100 μl of 1× PBS. Next, 50 μl of 75 pM Cy5-biotin-azide–labeled protein was added to the microfluidic chamber. After 2 min of incubation, unbound protein was washed away with 200 μl of PBS. Then, 50 μl of 5 nM donor-labeled aptamer strands in imaging buffer [2.5 mM protocatechuic acid (PCA) (Sigma-Aldrich: 37580), protocatechuate-3,4-dioxygenase (PCD) (0.155 U/μl; OYC Europe: 46852004), and 1 mM 6-hydroxy-2,5,7,8-tetramethylchroman-2-carboxylic acid (Trolox) (Sigma-Aldrich: 238813) in 1× PBS] was injected. All smFRET experiments were performed at room temperature (23 ± 1°C). See table S2 for the full list of aptamer sequences.

### Data analysis

Fluorescence signals are collected at 0.1-s exposure time. During the acquisition of the movie, the green laser is used to excite the Cy3 donor fluorophores. The fluorescence images were analyzed by a custom script written in Python (*35*, *36*). The script collects the individual intensity hotspots in the acceptor channel and pairs them with intensity hotspots in the donor channel, after which the time traces are extracted. The details of the automated detection of individual imager strand binding events from the fluorescence time traces are described elsewhere (*35*, *36*). Briefly, a two-state K-means clustering algorithm was applied on the sum of the donor and acceptor fluorescence intensities of individual molecules to determine an intensity threshold, with which the trace was divided into high- or low-intensity segments. The high-intensity segments that lasted for more than three consecutive frames were selected for further analysis. Average FRET efficiencies from each selected segment were used to build the FRET histogram. Populations in the FRET histogram are automatically classified using a Gaussian mixture model. The automated analysis code in Python is freely available at https://github.com/kahutia/transient_FRET_analyzer2. The kinetic rates and FRET efficiencies are determined using the following equations.

The dissociation rate (*k*_off_) is calculated as follows

$$k_{\text{off}}=\frac{1}{\tau_{b}}$$

where $\tau_{b}$ is the dwell time of the aptamer binding events. The association rate (*k*_off_) is calculated as follows

$$k_{\text{on}}=\frac{1}{\tau_{\mathrm{ub}}}\cdot c$$

where $\tau_{\mathrm{ub}}$ is the time in between aptamer binding events and *c* is the aptamer concentration. The dissociation constant *K*_d_ is calculated as follows

$$K_{d}=\frac{k_{\text{off}}}{k_{\text{on}}}$$

The apparent FRET efficiency is calculated as follows

$$\text{FRET}\;\left(E\right)=\frac{I_{A}}{I_{A}+I_{D}}$$

where $I_{A}$ is the intensity of the acceptor (Cy5) signal, and $I_{D}$ is the intensity of the donor (Cy3) signal.

### COMSOL simulation

Numerical simulations were carried out using COMSOL Multiphysics 6.1 on a system running Windows Server 2016 to model the mass transport and surface reaction kinetics of thrombin binding to an aptamer-functionalized QCM sensor. The governing equations and a comprehensive report of the simulation are provided in note S1. In summary, a QCM-D sensor cell (height = 0.64 mm, diameter = 11.1 mm), comprising the flow cell (inlet and outlet diameter = 1 mm) and sensor surface (bottom boundary of the chamber), was created. Physics interfaces for laminar flow, transport of diluted species, and a general form boundary partial differential equation (PDE) were used. The transport of diluted species interface modeled the diffusion and convection of thrombin in water, with the diffusion coefficient set to 8.76 × 10^−11^ m^2^/s as suggested in literature (*37*). The general form boundary PDE was used to describe the surface reaction kinetics, involving the binding and unbinding of thrombin to the aptamer. Boundary conditions included a specified thrombin concentration at the inlet, zero pressure at the outlet, and a weak form PDE for the sensor surface to model the flux of thrombin due to the surface interaction, based on a case study published in 2009 by Kwon *et al.* (*38*) A fine physics-controlled mesh for the respective modules was chosen, yielding 229,963 numbers of degrees of freedom (DOFs; plus 748,480 internal DOFs) (fig. S8). The aptamer concentration of 0.00023 mmol/m^2^ was maintained constant for all simulations. The concentration of thrombin (0.3 to 17 nM), flow rate (20 to 60 μl/min), and reaction rate constants *k*_on_ (10^6^ to 10^8^ M^−1^ s^−1^) and *k*_off_ (1 to 0.01 s^−1^) were varied independently. An additional simulation was conducted to incorporate the *k*_on_ and *k*_off_ values of the three tested aptamers obtained from the smFRET experiments, maintaining the flow rate constant at 40 μl/min and varying the target concentrations from 0.3 nM to17 nM. See tables S3 and S4 for a detailed list of COMSOL parameters used. The results from the simulations were processed using COMSOL’s built-in tools. This involved extracting and analyzing concentration profiles, velocity fields, and surface concentrations of the formed aptamer-protein complexes. Visualization was done using domain and boundary plots.

### QCM experiments

QCM characterization was conducted using a QE401 electronics unit with a QFM401 flow module and QCM QSX 301 gold-coated quartz crystals (AT-cut, 5 MHz) from Biolin Scientific (Gothenburg, Sweden). The sensor assembly protocol used in this study was adapted from a previously developed method by our group (*39*). A self-assembled monolayer (SAM) formation was performed outside the QCM flow module and all subsequent steps conducted inside (see details in the next paragraph). Data were recorded via QSoft (Biolin Scientific) software and processed in MATLAB (R2021b).

A SAM was created on a QCM chip gold surface by overnight incubation in a 10:90 mixture of 2 mM HSC_11_EG_6_OCH_2_COOH and 2 mM HSC_11_EG_5_OH (ProChimia Surfaces: TH 003-m11.n3-0.1, TH 001-m11.n1-0.2) dissolved in ethanol (99.5%; Sigma-Aldrich: 1.00983).

After SAM formation, the QCM chip was placed in the flow module, and PBS was flown over it for a 40-min equilibration (the flow rate was set to 45 ± 3 μl/min and kept constant for the entire experiment). Subsequently, a 100 mM 1:1 mixture of EDC and NHS (Sigma-Aldrich: 56485-1G, E7750-5G) in PBS was passed over the chip to activate the carboxylic groups of the SAM, forming an NHS ester. Following a short PBS washing step, the QCM flow module inlet tube was switched to the aptamer-containing solution (all aptamers were administered at 0.67 μM concentrations). To ensure no double-stranded aptamers were present, they were heated above their melting temperature to 90°C for 10 min before use. After flowing the aptamer solution, the inlet tube was switched back to PBS, concluding the sensor assembly (fig. S5). See table S2 for the full list of aptamer sequences. A control experiment was performed to confirm the passivation of the gold electrode on the QCM quartz, flowing thrombin solution (1.75 to 1690 nM) over the SAM surface without immobilized aptamers.

For sensitivity measurements, the assembled sensor was exposed to human alpha-thrombin in PBS at concentrations ranging from 0.06 to 16.5 nM for 20 min. To evaluate the sensor’s performance, baseline correction was performed and frequency values were averaged over 30 s both before the onset of a frequency drop and again after 5 min, to mitigate the influence of noise and QCM drift on the results. Transforming frequency to mass was achieved by using the quartz crystal sensitivity constant 17.7 ng Hz^−1^ cm^−2^ and the Sauerbrey equation. In addition, we compared the target dissociation within the first 5 min after stopping the target flow, normalizing the rates based on the total amount of captured thrombin at the end of the target flow. Linear regressions were used to indicate the initial trend in dissociation rates, thereby mitigating the influence of QCM drift and other delaying effects. For the regeneration experiments with NU172, three different solutions were tested: Milli-Q water, an 8 M urea buffer, and a NaOH regeneration buffer (25 mM NaOH and 0.5 M NaCl). After the initial thrombin capture sequence (16.9 nM), the regeneration buffer was flowed over the QCM chip for 5 min. This was followed by a 5- to 7-min flow of PBS to allow the aptamer to refold and stabilize the frequency before the second thrombin exposure. For additional validation experiments, the NU172 aptamer and 16.9 nM thrombin solutions were used, with flow rates varying between 25 and 58 μl/min. To enhance the visibility of QCM frequency changes in measurement plots, noise reduction was achieved by implementing a moving average (averaging window was set to 100 values).

### Statistics and reproducibility

No data were manually excluded from the analyses; however, data for some individual molecules have been rejected on quality, number of events (less than 10), and FRET efficiency during data processing and filtering, as described in the data analysis and aSyn mutant classification sections. These filtering steps can be reproduced using the code provided for those sections. The processed datasets were sufficiently large (~200 to 600 molecules) to ensure that distribution parameters could be determined with statistical significance. In classification tasks, the classifier was tested on sample data acquired from experiments different from those that produced training data, conducted on different days to avoid batch effects. Bootstrapping and 10-fold cross-validation were used to determine confidence intervals and prediction intervals, respectively. QCM results were analyzed using MATLAB, applying a baseline correction. All experiments were conducted at least in triplicate unless noted otherwise. Experiments demonstrating aptamer immobilization of less than 1% were excluded from further analysis. If the aptamer immobilization efficiency fell below 15% or was above 50%, an additional test was performed to ensure that the grafting density of the aptamers was not influencing the result. Statistical analysis was based on *t* tests within a 95% confidence interval.

## Supplementary Materials

### This PDF file includes:

Figs. S1 to S9 Tables S1 to S5 Note S1 References
