# Supplementary material for: Decoding aptamer-protein binding kinetics for continuous biosensing using single-molecule techniques
Source: Sci Adv. 2025 Feb 14;11(7):eads9687. doi: 10.1126/sciadv.ads9687 (PMC11827629; doi:10.1126/sciadv.ads9687)
Supplement: Supplementary file 1 — Figs. S1 to S9 Tables S1 to S5 Note S1 References [file sciadv.ads9687_sm.pdf]

Science Advances
AAAS

Supplementary Materials for

Decoding aptamer-protein binding kinetics for continuous biosensing using single-molecule techniques

Mike Filius *et al.*

Corresponding author: Alina Y. Rwei, a.y.rwei@tudelft.nl; Chirlmin Joo, c.joo@tudelft.nl

Sci. Adv. **11**, eads9687 (2025)
DOI: 10.1126/sciadv.ads9687

This PDF file includes:

Figs. S1 to S9
Tables S1 to S5
Note S1
References

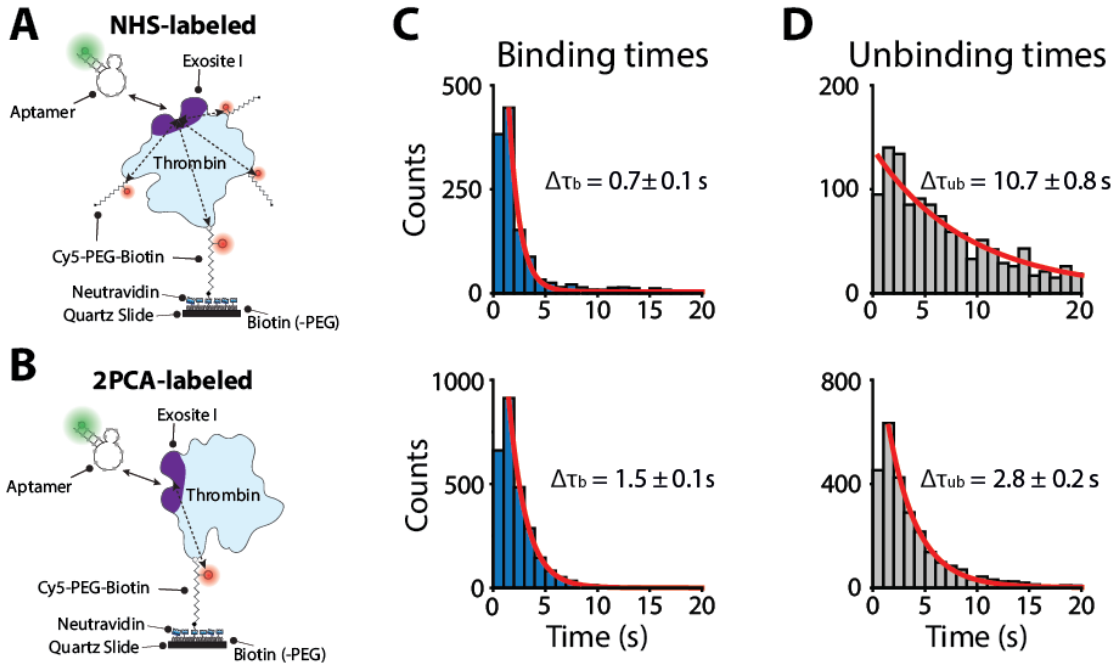

Figure S1: Single-molecule kinetic analysis of NHS- and 2PCA-labeled thrombin. (A and B) Schematic representation of the single-molecule FRET assay. The NHS [A] and 2PCA [B] acceptor (Cy5)-labeled thrombin proteins are immobilized on a PEGylated quartz slide through biotin-neutravidin conjugation. Binding of the donor (Cy3)-labeled aptamer to the protein target yields a FRET signal. (**C and D**) The binding-(τ_b, panel C) and unbinding (τ_{ub}, panel D) dwell time distributions are plotted for aptamer HD1 binding to NHS- (top) and 2PCA-labeled (bottom) thrombin and are fitted with a single exponential decay curve. The dwell times are reported as the mean ± the standard deviation of three independent experiments.

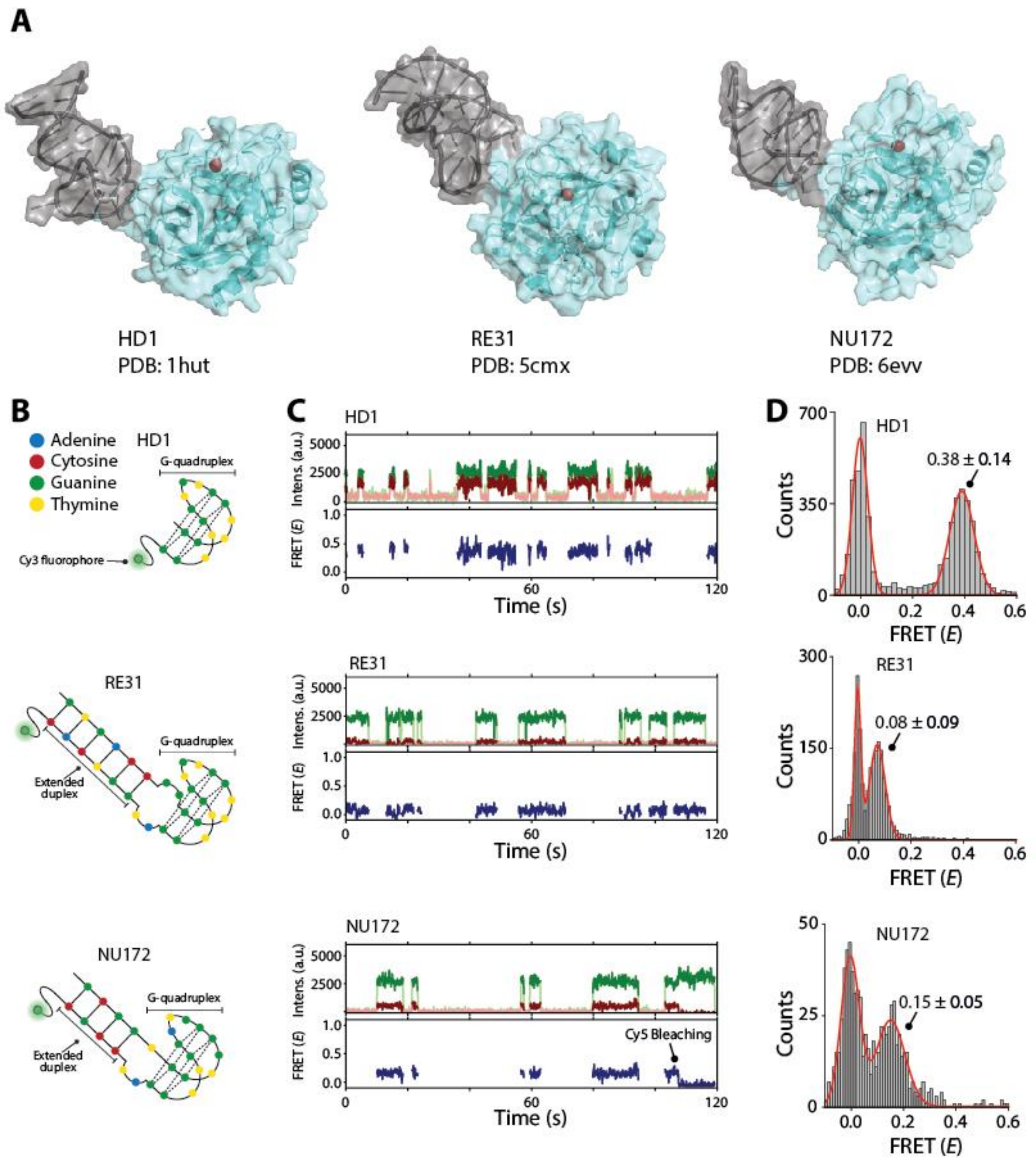

Figure S2: Single-molecule FRET for localisation of different aptamers on individual thrombin molecules. (A) Crystal structures showing the binding location of aptamers HD1 (left), RE31 (middle), and NU172 (right) with the thrombin target protein (blue structure). The different aptamers target the same Exosite I of the thrombin target protein. **(B)** Schematic representation of the different aptamers used. Aptamers RE31 and NU172 are stabilized analogues of HD1 by the addition of duplex domains to the G-quadruplex of HD1. The length of the duplex and the sequence of the aptamer are different for RE31 and NU172. **(C)** Representative time traces showing short and repetitive aptamer binding to the thrombin protein. Each of the binding events yields a FRET signal reporting on the relative binding location of the aptamer to the N terminus reference point. **(D)** The single molecule FRET distributions are made from the FRET efficiency determined for each binding event. The distributions were fitted with a Gaussian and the FRET efficiency is reported as the mean ± the FWHM of the gaussian fits (HD1, n=3117 events, RE31, n=2058 events, NU172, n=750 events).

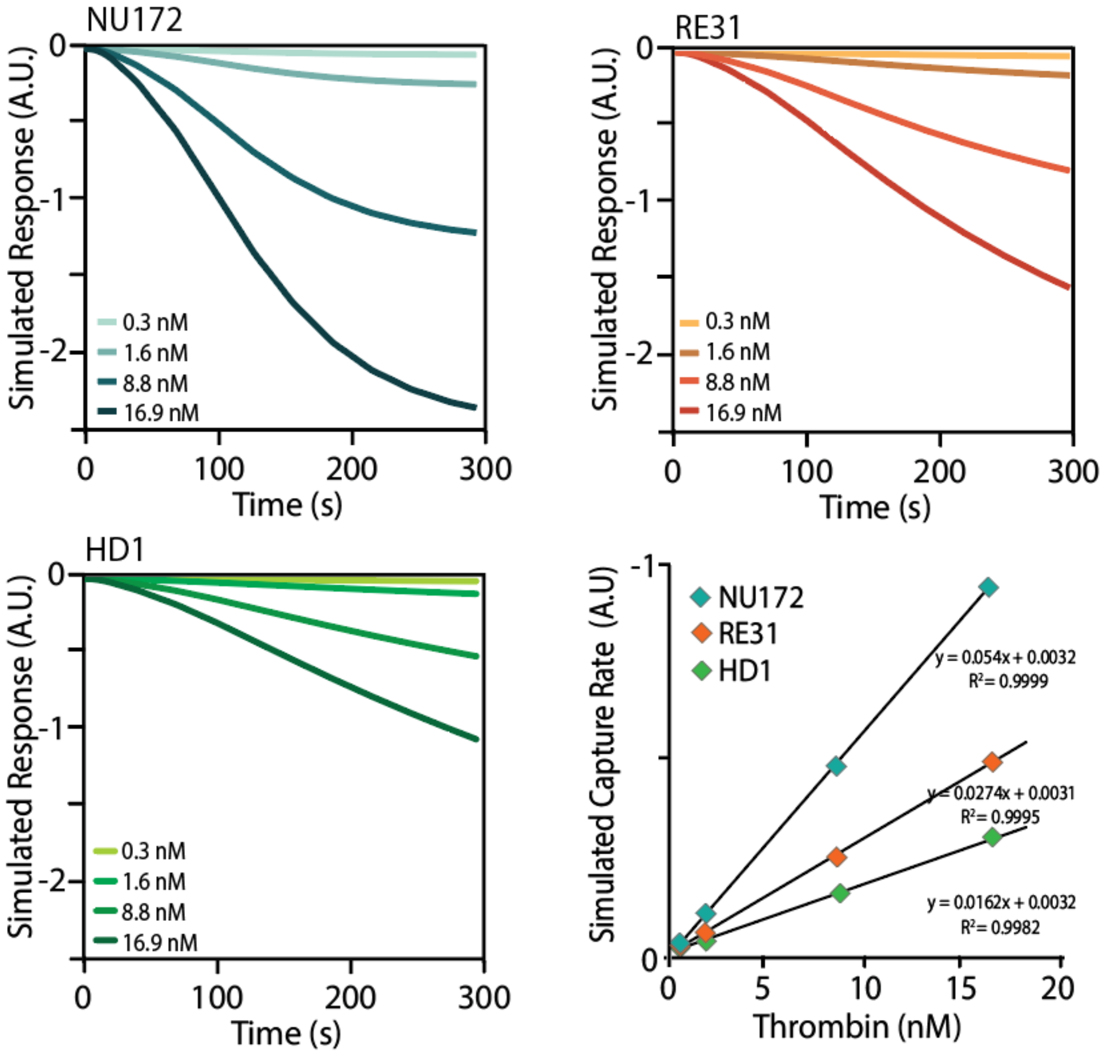

Figure S3. Simulated sensor response. Simulation of the sensor response with the k_{on} and k_{off} values of the single molecule data for NU172, RE31, and HD1, with the following parameters: flow rate of 40 µL/min and thrombin concentration of 17 nM. The simulated capture response was normalized, revealing that NU172 exhibited the highest capture rate, approximately twice as high as RE31, while HD1 showed the lowest capture of the three tested aptamers.

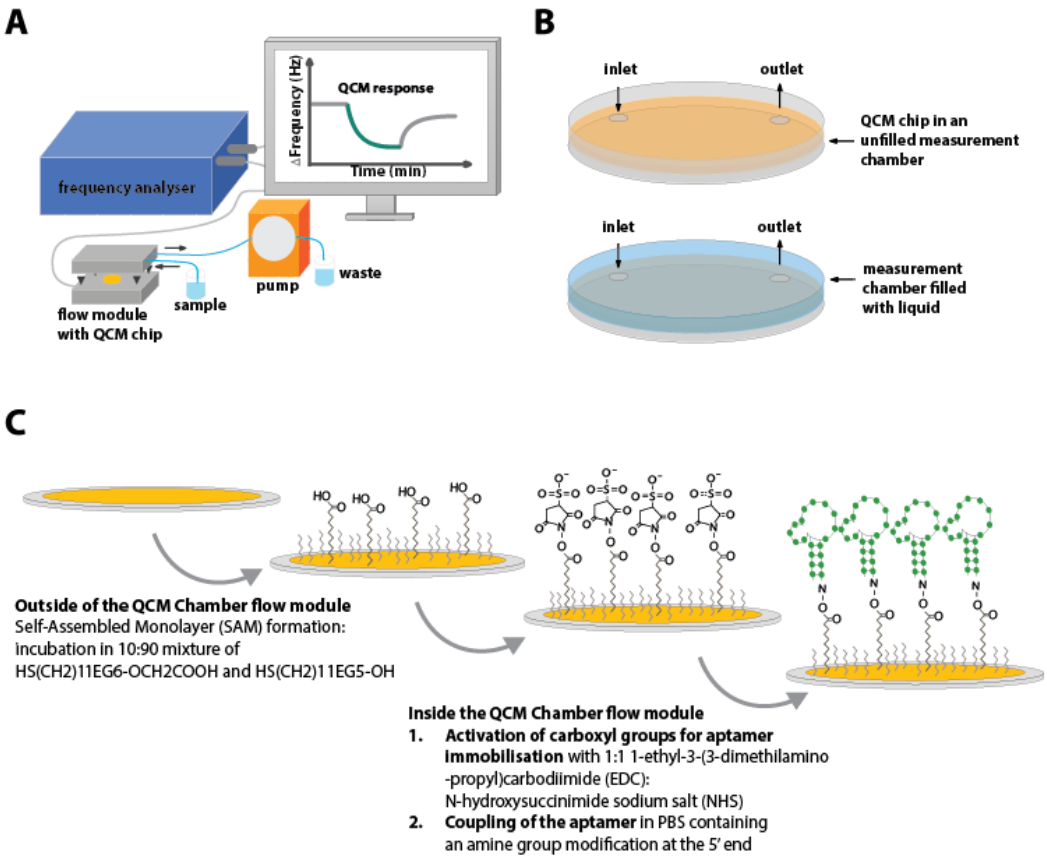

Figure S4: Experimental set-up and Schematic workflow of QCM sensor assembly. A) Schematic of QCM set-up; B) Schematic of QCM empty and filled measurement chamber including the QCM chip. C) Schematic of sensor assembly outside and inside the QCM measurement chamber.

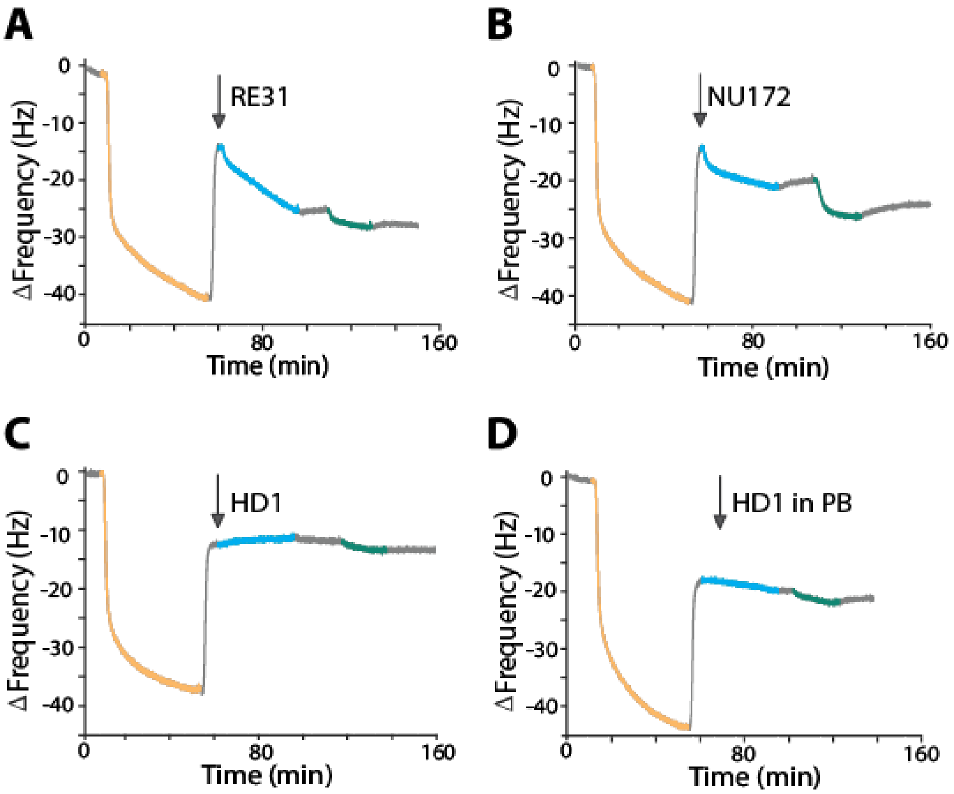

Figure S5: Aptamer immobilization. Exemplary QCM measurement results for all three aptamers. Aptamer immobilisation during in-situ sensing layer assembly was followed continuously and adsorbed mass (Δm) on the sensor surface results in a frequency decrease (Δf), as described by the Sauerbrey equation. The assembly inside the measurement chamber begins with the EDC:NHS activation (yellow trace), followed by a brief rinsing with PBS, and subsequent aptamer immobilization (blue trace, start indicated by arrow). The immobilization of aptamers RE31 (**A**) and NU 172 (**B**) resulted in a significant frequency decrease, with an average immobilization efficiency—defined as the ratio of available COOH groups to immobilized aptamer molecules—of 19% for both aptamers. In contrast, the immobilization of HD1 (**C**) could not be conclusively confirmed. Despite the frequency decrease during target capture indicating successful immobilization, the lack of reproducibility led to the exclusion of HD1 from further evaluation. We hypothesized that in the presence of potassium ions, HD1 predominantly adopts a folded structure, which may interfere with its immobilization. Without this stabilizing K^+ ion, HD1 is reported to have lower folding efficiency and slower folding kinetics, potentially facilitating immobilization.(*40*) We conducted a control experiment in a potassium-free phosphate buffer (PB) system (**D**), and we observed an immobilization efficiency of approximately 12 ± 2 % indicating that indeed the unfolded HD1 can be immobilized contrarily as opposed to the folded form. However, as the tertiary structure significantly influences the aptamer's affinity, we chose not to include this data in the manuscript to avoid confounding effects from buffer-induced structural changes.

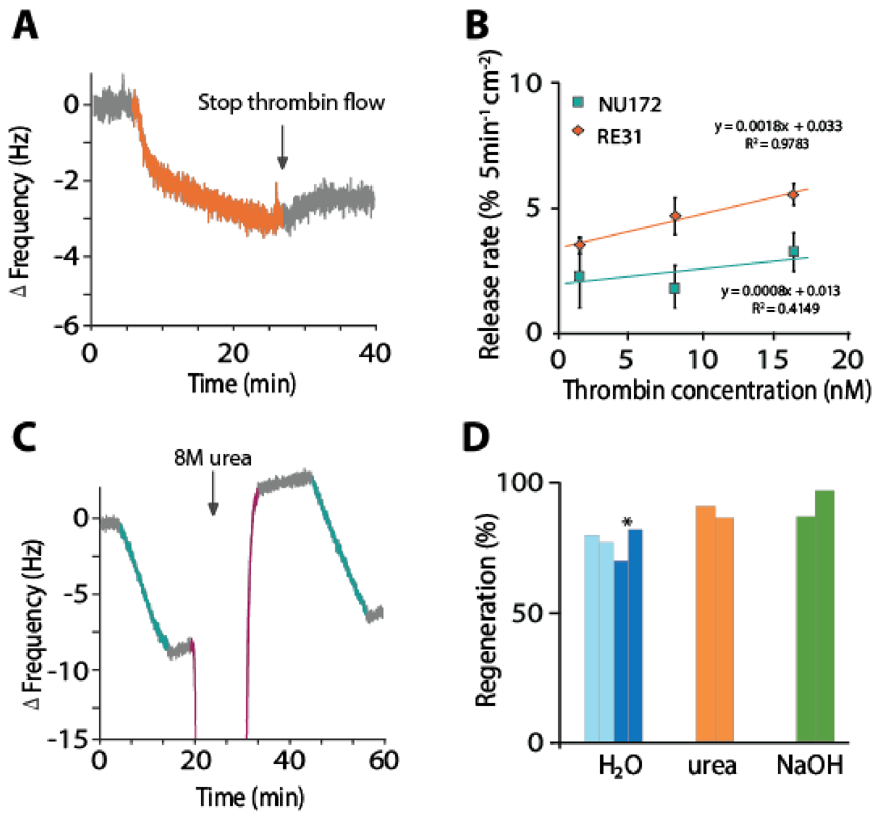

Figure S6: Dissociation behaviour of aptamers. (A) Exemplary experimental result of RE31: arrow indicates beginning of dissociation phase **(B)** Evaluation results of target dissociation within the first 5 minutes after stopping the target flow. The rates were normalized based on the amount of captured thrombin at the end of the target flow to eliminate the influence of thrombin concentrations. Linear regressions were employed to indicate the trends in dissociation rates, thereby mitigating the influence of QCM drift and other delaying effects, such as the retention-rebinding effect, which are expected to become more significant in the later stages of the experiment. The dissociation behaviour observed in the QCM experiments demonstrated slower dissociation rates for the NU 172 aptamer in comparison to RE31. We only observed a statistically significant difference in dissociation behaviour between the two aptamers only at the highest thrombin concentration (16.5 nM). Here, RE31 demonstrated an approximately doubled desorption rate (thrombin release 6.3% ± 0.6% per 5 min) compared to NU172 (thrombin release 3.1% ± 1.05% per 5 min) (t-test: $t(6) = 3.71$, $P = 0.0340$, 95% confidence interval). For both aptamers, the signal did not return to the initial baseline, suggesting a rebinding-retention(*41*, *42*) effect where thrombin remains bound for an extended time period due to re-binding to nearby aptamers. This explains the reduced sensor response upon re-exposure to the target, with response ranging from 24% to 46% of the initial response, indicating partial surface saturation **(C)** Exemplary NU172 regeneration experiment using 8M urea buffer (highlighted in purple) in between exposure to the thrombin (turquoise trace) and PBS rinsing (grey trace). **(D)** Results of NU172 and RE31 (labelled with *) regeneration experiments with MilliQ water resulting in similar recovery of 79 ± 2% and 76 ± 8% of the initial signal. A further optimization in regeneration using the NU172 aptamer improved to approximately 89 ± 3% with an 8 M urea buffer and reached 91 ± 7% recovery after using a NaOH regeneration buffer (n=2). After two consecutive regeneration cycles, a significant decrease in the signal was observed likely due to degradation or detachment of the sensing layer. Optimizing the regeneration procedure including fine-tuning length of regeneration period and concentration of regeneration buffers can enhance the reusability of QCM assays as previously shown. (*43*)

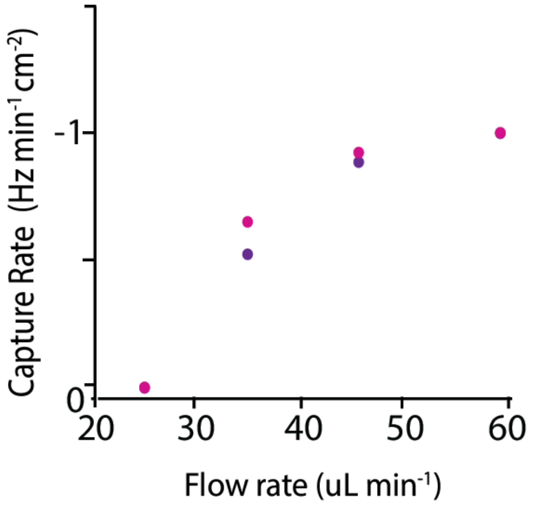

Figure S7. Analyte capture dependency on flow rate in QCM chamber. Increasing the flow rate from 25 µL/min to 58 µL/min resulted in an increasing thrombin capture rate by the NU172 aptamer, which indicates a mass transfer limitation of the target to the substrate surface. As observed in the QCM simulation, at a lower flow rate (25 uL/min) the capture rate substantially dropped to only 4 ± 1 % compared to the signal at 45 uL/min (n=2). Notably, the flow rate of 58 µL/min did not produce a significant increase in the signal, suggesting that effective mass transfer has been achieved with the higher flowrate values.

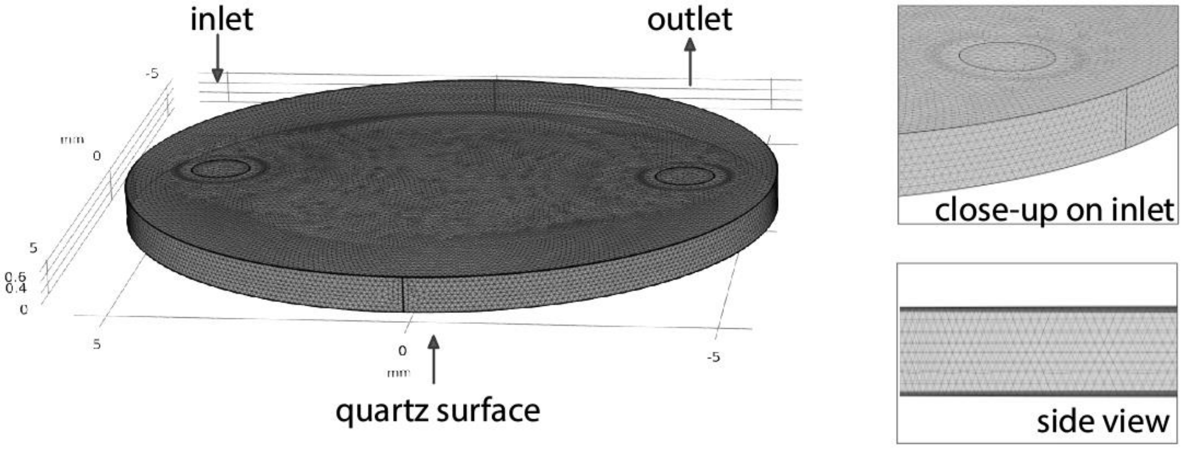

Figure S8. COMSOL simulation mesh. A fine physics-controlled mesh was selected for the COMSOL simulation. Additionally, close-up views of the inlet and a side perspective are provided.

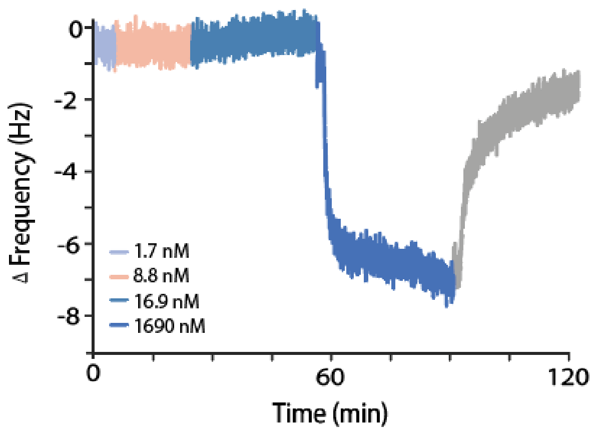

Figure S9. QCM control experiment. Thrombin adsorption on the self-assembled monolayer-modified quartz electrode showed no non-specific adsorption within the concentration range investigated in this study, indicating effective passivation. Only at a concentration of 1.6 µM did thrombin cause a frequency decrease of approximately 7 Hz.

Supplementary Table 1: Kinetic overview of thrombin binding aptamers.

	Our work			Literature	
**Aptamers**	k_{off} (s^{-1})	k_{on} (10^8 M^{-1} s^{-1})	K_D (nM)	K_D (nM)	**Reference**
**HD1**	0.7 ± 0.1	0.7 ± 0.1	9.2 ± 0.9	7.1 ± 0.2	Muller et al.(*44*)
**RE31**	0.3 ± 0.1	1.1 ± 0.1	2.9 ± 0.1	1.4 ± 0.1	Kotkowiak et al. (*16*)
**NU172**	0.1 ± 0.1	0.2 ± 0.1	3.3 ± 0.8	3.2 ± 0.8	Trapaidze et al. (*45*)

Supplementary Table 2: Aptamer Sequences

**DNA Strand**	**Sequence (5' - 3')**	**Modification**	**Supplier**
HD1 single molecule exp.	TTTTTTTGGTTGGTGTGGTTGG	5' Cy3	Ella Biotech (GmbH)
RE31 single molecule exp.	TTTTTTTGTGACGTAGGTTGGTGTGGTTGGGGCGTCAC	5' Cy3	Ella Biotech (GmbH)
NU172 singe molecule exp.	TTTTTTTCGCCTAGGTTGGGTAGGGTGGTGGCG	5' Cy3	Ella Biotech (GmbH)
HD1 QCM exp.	TTTTTTTGGTTGGTGTGGTTGG	5' Amino C6	Ella Biotech (GmbH)
RE31 QCM exp.	TTTTTTTGTGACGTAGGTTGGTGTGGTTGGGGCGTCAC	5' Amino C6	Ella Biotech (GmbH)
NU172 QCM exp.	TTTTTTTCGCCTAGGTTGGGTAGGGTGGTGGCG	5' Amino C6	Ella Biotech (GmbH)

Supplementary Table 3: COMSOL parameters

Name	Value	Description
r_chamber	0.00555 m	Radius QCM chamber
h_chamber	6.4E−4 m	Height QCM chamber
r_in	5E−4 m	Radius inlet/outlet
h_in	1E−4 m	Height inlet/outlet
Q	1E−9 m^3/s	Flow rate
D_B	8.76E−11 m^2/s	Diffusion coefficient of thrombin
cB	1.7E-5 mol/m^3	Concentration thrombin
cA	2.3E−7 mol/m^2	Concentration immobilized aptamers
kf	1.1E5 $m^3/(s·mol)$	Forward reaction rate constant (k_{on} rate)
kr	0.01 1/s	Reverse reaction rate constant (k_{off} rate)
Ds	9.2E−15 m^2/s	Surface diffusivity aptamers immobilized via a thiol group (from (*46*))

Supplementary Table 4: Variation range of COMSOL parameters

Parameter name	Parameter value list	Parameter unit
Q	20, 40, 60	uL min^{-1}
cB	0.3, 1.7, 8.75, 16.95	nmol l^{-1}
kf	$1*10^6$, $1*10^7$, $1*10^8$	M^{-1} s^{-1}
kr	1, 0.1, 0.01	s^{-1}

Supplementary Table 5: Measurement data sensitivity studies

Thrombin Concentration (nM)	NU172 Capture rate (Hz min^{-1} cm^{-2})			RE31 Capture rate (Hz min^{-1} cm^{-2})		
0.3	0.065	0.033	0.040	-0.018	0.008	-0.008
1.7	0.181	0.124	0.195	0.060	0.094	0.101
8.5	0.356	0.518	0.469	0.159	0.174	0.256
16.9	0.948	1.073	1.041	0.462	0.367	0.5787

Supplementary Note 1. COMSOL Simulation Report

The computational simulations were conducted using COMSOL Multiphysics version 6.1 on a Windows Server 2016 system. The simulation setup utilized the SI unit system, with key global parameters given in **Supplementary Table 2.**

For thrombin – a protein with a molecular weight of 36 kDa, the diffusion coefficient can be estimated using the Stokes-Einstein equation

$$D = \frac{k_B T}{6\pi\eta R_H}$$

With D as the diffusion coefficient, k_B the Boltzmann constant, T the absolute temperature (Kelvin), η the dynamic viscosity of the solvent (water) (0.89×10^{-3} Pa·s), R_H is the hydrodynamic radius of the protein.

To estimate the hydrodynamic radius R_H, we used a common approximation based on empirical relationships that relate the molecular weight (M) of the protein to its radius.

$$R_H \approx 0.066 \times M^{\frac{1}{3}} \approx 0.066 \times 36000^{\frac{1}{3}} \approx 2.22\, nm$$

That allowed for the estimation of the diffusion coefficient as follows:

$$D = \frac{1.38 \times 10^{-23} \ * 298}{6\pi \times \ 0.89 \times 10^{-3} \times 2.22 \times 10^{-9}} = \frac{4.11 \times 10^{-11}}{3.71 \times 10^{-11}} = 1.11 \times 10^{-11} m\backslash s$$

The diffusion coefficient reported in the literature was in good agreement with the estimation above and was further utilized for the simulation.

The simulation geometry consisted of a cylindrical chamber with a radius of 5.55 mm and a height of 0.64 mm. The inlet and outlet were modelled as smaller cylinders created on the top surface of the measurement chamber, each with a radius of 0.5 mm (**Fig. 3b**).

The system's fluid flow was modeled using the laminar flow module as a Newtonian, incompressible fluid, governed by the Navier-Stokes equation.

$$\rho(\mathbf{u} \cdot \nabla)\mathbf{u} = \nabla \cdot [-p\mathbf{I} + \mathbf{K}] + \mathbf{F}$$

The continuity equation for incompressible flow, was applied to ensure mass conservation.

$$\rho \nabla \cdot \mathbf{u} = 0$$

The fluid flow was approximated as a steady-state process.

The system included two species: the immobilized aptamer species (A) and the mobile Thrombin (B). The transport of Thrombin was modelled using the Transport of diluted species module and its predefined mass transfer equation:

$$\frac{\partial c_i}{\partial t} + \nabla \cdot \mathbf{J}_i + \mathbf{u} \cdot \nabla c_i = R_i$$
$$\mathbf{J}_i = -D_i \nabla c_i$$

The velocity field "u" was determined by solving the study on laminar flow. The reaction and adsorption of biomolecules occurred on the upper surface of the QCM crystal's gold electrode. The reaction kinetics were modelled using a pseudo-first-order reaction, described by the equation:

$$\frac{\partial c_{AB}}{\partial t} = k_f c_B (c_A - c_{AB}) - k_r c_{AB}$$

with c_{AB} as the molar concentration of the bound complex (mol/m^2), the surface concentration of the thrombinc_B, the initial concentration of the immobilized aptamer c_A, the forward reaction rate constant k_f, and the reverse reaction rate constant k_r. The initial condition for the bound complex concentration was set to zero, $c_{AB} = 0$, at the start of the simulation.

The boundary conditions for the inlet were defined as the concentration of the inflowing thrombin concentration c_B. After 100 seconds a smoothed step (smoothed zone 40) function was used to initiate rinsing of the flow chamber with water and decrease the thrombin concentration to 0 nM.

On the active sensor surface, the defined boundary condition couples the rate of the reaction at the surface with the thrombin flux:

$$-D\nabla c_B = k_f c_B(c_A - c_{AB}) - k_r c_{AB}$$

The mesh used in the model resulted in approximately 230000 degrees of freedom (**Fig. S8**).

The simulation was run over a period of 300 seconds. The simulation study focused on the effect of flow rate, concentration of thrombin, and the forward and reverse reaction rate constants (k_f and k_r, respectively), the concentration of the thrombin c_B, The range of values used in this study, as outlined in **Supplementary Table 3**, were chosen to reflect those typically encountered in real-world QCM binding experiments. However, the concentration of c_A was set to a high concentration to avoid the need for extremely fine-meshed domains.

REFERENCES AND NOTES

1. J. Kim, A. S. Campbell, B. E.-F. de Ávila, J. Wang, Wearable biosensors for healthcare monitoring. *Nat. Biotechnol.* **37**, 389–406 (2019).

2. H. C. Ates, P. Q. Nguyen, L. Gonzalez-Macia, E. Morales-Narváez, F. Güder, J. J. Collins, C. Dincer, End-to-end design of wearable sensors. *Nat. Rev. Mater.* **7**, 887–907 (2022).

3. B. S. Ferguson, D. A. Hoggarth, D. Maliniak, K. Ploense, R. J. White, N. Woodward, K. Hsieh, A. J. Bonham, M. Eisenstein, T. E. Kippin, K. W. Plaxco, H. T. Soh, Real-time, aptamer-based tracking of circulating therapeutic agents in living animals. *Sci. Transl. Med.* **5**, 213ra165 (2013).

4. T. Adachi, Y. Nakamura, Aptamers: A review of their chemical properties and modifications for therapeutic application. *Molecules* **24**, 4229 (2019).

5. M. Liss, B. Petersen, H. Wolf, E. Prohaska, An aptamer-based quartz crystal protein biosensor. *Anal. Chem.* **74**, 4488–4495 (2002).

6. O. A. Alsager, K. M. Alotaibi, A. M. Alswieleh, B. J. Alyamani, Colorimetric aptasensor of vitamin D3: A novel approach to eliminate residual adhesion between aptamers and gold nanoparticles. *Sci. Rep.* **8**, 12947 (2018).

7. H.-J. Chen, R. L. C. Chen, B.-C. Hsieh, H.-Y. Hsiao, Y. Kung, Y.-T. Hou, T.-J. Cheng, Label-free and reagentless capacitive aptasensor for thrombin. *Biosens. Bioelectron.* **131**, 53–59 (2019).

8. Z. Ding, S. Yang, J. Wang, Z. Zhao, H. Xu, Z. Chen, Z. Liu, Y. Wang, J. Bao, K. Chang, M. Chen, Rolling circle amplification/G-quadruplex-based dual-signal ratiometric electrochemical aptasensor for ultrasensitive detection of pathogenic bacteria. *ChemElectroChem* **10**, e202300257 (2023).

9. M. Ilgu, M. Nilsen-Hamilton, Aptamers in analytics. *Analyst* **141**, 1551–1568 (2016).

10. M. Ilgu, D. B. Fulton, R. M. Yennamalli, M. H. Lamm, T. Z. Sen, M. Nilsen-Hamilton, An adaptable pentaloop defines a robust neomycin-B RNA aptamer with conditional ligand-bound structures. *RNA* **20**, 815–824 (2014).

11. S. Cai, J. Yan, H. Xiong, Y. Liu, D. Peng, Z. Liu, Investigations on the interface of nucleic acid aptamers and binding targets. *Analyst* **143**, 5317–5338 (2018).

12. P. Schuck, H. Zhao, The role of mass transport limitation and surface heterogeneity in the biophysical characterization of macromolecular binding processes by SPR biosensing, in *Surface Plasmon Resonance: Methods and Protocols*, N. J. Mol, M. J. E. Fischer, Eds. (Humana Press, 2010), pp. 15–54; https://doi.org/10.1007/978-1-60761-670-2_2).

13. B. Deng, Y. Lin, C. Wang, F. Li, Z. Wang, H. Zhang, X.-F. Li, X. C. Le, Aptamer binding assays for proteins: The thrombin example—A review. *Anal. Chim. Acta* **837**, 1–15 (2014).

14. I. Russo Krauss, V. Spiridonova, A. Pica, V. Napolitano, F. Sica, Different duplex/quadruplex junctions determine the properties of anti-thrombin aptamers with mixed folding. *Nucleic Acids Res.* **44**, 983–991 (2016).

15. C. Platella, C. Riccardi, D. Montesarchio, G. N. Roviello, D. Musumeci, G-quadruplex-based aptamers against protein targets in therapy and diagnostics. *Biochim. Biophys. Acta* **1861**, 1429–1447 (2017).

16. W. Kotkowiak, J. Wengel, C. J. Scotton, A. Pasternak, Improved RE31 analogues containing modified nucleic acid monomers: Thermodynamic, structural, and biological effects. *J. Med. Chem.* **62**, 2499–2507 (2019).

17. J. Nick Taylor, Q. Darugar, K. Kourentzi, R. C. Willson, C. F. Landes, Dynamics of an anti-VEGF DNA aptamer: A single-molecule study. *Biochem. Biophys. Res. Commun.* **373**, 213–218 (2008).

18. T. Xia, J. Yuan, X. Fang, Conformational dynamics of an ATP-binding DNA aptamer: A single-molecule study. *J. Phys. Chem. B* **117**, 14994–15003 (2013).

19. M. Filius, R. van Wee, C. de Lannoy, I. Westerlaken, Z. Li, S. H. Kim, C. de Agrela Pinto, Y. Wu, G.-J. Boons, M. Pabst, D. de Ridder, C. Joo, Full-length single-molecule protein fingerprinting. *Nat. Nanotechnol.* **19**, 652–659 (2024).

20. J. I. Macdonald, H. K. Munch, T. Moore, M. B. Francis, One-step site-specific modification of native proteins with 2-pyridinecarboxyaldehydes. *Nat. Chem. Biol.* **11**, 326–331 (2015).

21. A. Avino, C. Fabrega, M. Tintore, R. Eritja, Thrombin binding aptamer, more than a simple aptamer: Chemically modified derivatives and biomedical applications. *Curr. Pharm. Des.* **18**, 2036–2047 2012.

22. M. T. Murray, S. D. Wetmore, Unlocking precision in aptamer engineering: A case study of the thrombin binding aptamer illustrates why modification size, quantity, and position matter. *Nucleic Acids Res.* **52**, 10823–10835 (2024).

23. R. Troisi, F. Sica, Structural overview of DNA and RNA G-quadruplexes in their interaction with proteins. *Curr. Opin. Struct. Biol.* **87**, 102846 (2024).

24. R. Troisi, V. Napolitano, V. Spiridonova, I. Russo Krauss, F. Sica, Several structural motifs cooperate in determining the highly effective anti-thrombin activity of NU172 aptamer. *Nucleic Acids Res.* **46**, 12177–12185 (2018).

25. E. Ismail, Y. Liu, Y. Wang, S. Y. Tafti, X. F. Zhang, X. Cheng, Aptamer-based biotherapeutic conjugate for shear responsive release of Von Willebrand factor A1 domain. *Nanoscale* **17**, 1246–1259 (2025).

26. P. Skládal, C. dos Santos Riccardi, H. Yamanaka, P. I. da Costa, Piezoelectric biosensors for real-time monitoring of hybridization and detection of hepatitis C virus. *J. Virol. Methods* **117**, 145–151 (2004).

27. M. Domsicova, S. Kurekova, A. Babelova, K. Jakic, I. Oravcova, V. Nemethova, F. Razga, A. Breier, M. Gal, A. Poturnayova, Advancements in chronic myeloid leukemia detection: Development and evaluation of a novel QCM aptasensor for use in clinical practice. *Biochem. Biophys. Rep.* **39**, 101816 (2024).

28. D. Proudnikov, A. Mirzabekov, Chemical methods of DNA and RNA fluorescent labeling. *Nucleic Acids Res.* **24**, 4535–4542 (1996).

29. K. Lang, J. W. Chin, Bioorthogonal reactions for labeling proteins. *ACS Chem. Biol.* **9**, 16–20 (2014).

30. R. Van Wee, M. Filius, C. Joo, Completing the canvas: Advances and challenges for DNA-PAINT super-resolution imaging. *Trends Biochem. Sci.* **46**, 918–930 (2021).

31. A. M. Downs, K. W. Plaxco, Real-time, in vivo molecular monitoring using electrochemical aptamer based sensors: Opportunities and challenges. *ACS Sens.* **7**, 2823–2832 (2022).

32. H. Yoo, H. Jo, S. S. Oh, Detection and beyond: Challenges and advances in aptamer-based biosensors. *Mater. Adv.* **1**, 2663–2687 (2020).

33. S. D. Chandradoss, A. C. Haagsma, Y. K. Lee, J.-H. Hwang, J.-M. Nam, C. Joo, Surface passivation for single-molecule protein studies. *J. Vis. Exp.* 10.3791/50549, 50549 (2014).

34. M. Filius, T. J. Cui, A. N. Ananth, M. W. Docter, J. W. Hegge, J. van der Oost, C. Joo, High-speed super-resolution imaging using protein-assisted DNA-PAINT. *Nano Lett.* **20**, 2264–2270 (2020).

35. M. Filius, S. H. Kim, I. Severins, C. Joo, High-resolution single-molecule FRET via DNA eXchange (FRET X). *Nano Lett.* **21**, 3295–3301 (2021).

36. S. H. Kim, H. Kim, H. Jeong, T.-Y. Yoon, Encoding multiple virtual signals in DNA barcodes with single-molecule FRET. *Nano Lett.* **21**, 1694–1701 (2021).

37. G. P. Agarwal, J. G. Gallagher, K. C. Aune, C. D. Armeniades, Investigation of the aggregation and activation of prothrombin using quasi-elastic light scattering. *Biochemistry* **16**, 1865–1870 (1977).

38. H. J. Kwon, C. K. Bradfield, B. T. Dodge, G. S. Agoki, Study of simultaneous fluid and mass adsorption model in the QCM-D sensor for characterization of biomolecular interactions, in *Proceedings of the COMSOL Conference 2009 Boston* (COMSOL, 2009).

39. P. Zhu, V. A. Papadimitriou, J. E. van Dongen, J. Cordeiro, Y. Neeleman, A. Santoso, S. Chen, J. C. T. Eijkel, H. Peng, L. I. Segerink, A. Y. Rwei, An optical aptasensor for real-time quantification of endotoxin: From ensemble to single-molecule resolution. *Sci. Adv.* **9**, eadf5509 (2023).

40. B. I. Kankia, L. A. Marky, Folding of the thrombin aptamer into a G-quadruplex with Sr^{2+}: Stability, heat, and hydration. *J. Am. Chem. Soc.* **123**, 10799–10804 (2001).

41. T. J. Silhavy, S. Szmelcman, W. Boos, M. Schwartz, On the significance of the retention of ligand by protein. *Proc. Natl. Acad. Sci. U.S.A.* **72**, 2120–2124 (1975).

42. H. Zhao, L. F. Boyd, P. Schuck, Measuring protein interactions by optical biosensors. *Curr. Protoc. Protein Sci.* **88**, 20.2.1-20.2.25 (2017).

43. F. Beyazit, M. Y. Arica, I. Acikgoz-Erkaya, C. Ozalp, G. Bayramoglu, Quartz crystal microbalance–based aptasensor integrated with magnetic pre-concentration system for detection of Listeria monocytogenes in food samples. *Microchim. Acta* **191**, 235 (2024).

44. J. Müller, D. Freitag, G. Mayer, B. Pötzsch, Anticoagulant characteristics of HD1-22, a bivalent aptamer that specifically inhibits thrombin and prothrombinase. *J. Thromb. Haemost.* **6**, 2105–2112 (2008).

45. A. Trapaidze, J.-P. Hérault, J.-M. Herbert, A. Bancaud, A.-M. Gué, Investigation of the selectivity of thrombin-binding aptamers for thrombin titration in murine plasma. *Biosens. Bioelectron.* **78**, 58–66 (2016).

46. L. L. Rouhana, M. D. Moussallem, J. B. Schlenoff, Adsorption of short-chain thiols and disulfides onto gold under defined mass transport conditions: Coverage, kinetics, and mechanism. *J. Am. Chem. Soc.* **133**, 16080–16091 (2011).
